# Supplementary material for: The Evolution and Characterization of the RNA Interference Pathways in Lophotrochozoa
Source: Genome Biol Evol. 2024 May 7;16(5):evae098. doi: 10.1093/gbe/evae098 (PMC11114477; doi:10.1093/gbe/evae098)
Supplement: evae098_Supplementary_Data [file evae098_supplementary_data.zip › Supplementary_materials.zip!/selected_miRNAs/NoGe_selected_miRNAs/known_miRNA_families_pdf_files/NMRB01000087.1_Notospermus_geniculatus_strain_Ushimado_scaffold87,_whole_genome_shotgun_sequence_3154.pdf]

Provisional ID : NMRB01000087.1_Notospermus_geniculatus_strain_Ushimado_scaffold87,_whole_genome_shotgun_sequence_3154
Score total : 2852.2
Score for star read(s) : 3.9
Score for read counts : 2843.4
Score for mfe : 1.8
Score for randfold : 0
Score for cons. seed : 3
Total read count : 5589
Mature read count : 5583
Loop read count : 0
Star read count : 6

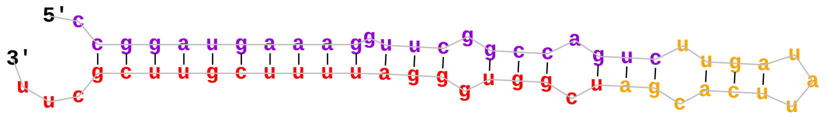

Genome coordinates: NMRB01000087.1 27092-27150

pre-miRNA sequence:
CCGGAUGAAAGGUUCGGCCAGUCCCGGAUGAAAGGUUCGGCCAGUCUUGAUAUUCACGAUCGGUGGGAUUUUCGUUCGCUU

Mature sequence: UCGGUGGGAUUUUCGUUCGCUU

Aligned mature sequence of the *N. geniculatus* pre-miRNA with the top 5 hits from the BLAST search against the pre-miRNA mirGeneDB set:

```
                                ******************* *
    Nge_Mir-278                 ucggugggauuuucguucgcuu
    Efe-Mir-278-P2_3p           ucggugggauuuucguucguuc
    Lan-Mir-278_3p              ucggugggauuuucguucgguc
```
